# Dual effect of Sapogenins extracted from *Spirulina platensis* on telomerase activity in two different cell lines

**DOI:** 10.22099/mbrc.2020.38230.1537

**Published:** 2021-03

**Authors:** Mahboobeh Akbarizare, Hamideh Ofoghi, Mahnaz Hadizadeh

**Affiliations:** Iranian Research Organization for Science and Technology (IROST), Tehran, Iran

**Keywords:** Spirulina platensis, Sapogenins, Telomerase activator, Telomerase inhibitor

## Abstract

*Spirulina platensis* is a photosynthetic filamentous, edible cyanobacterium that is known as a superfood. In this study, sapogenins were extracted from the *spirulina* and the effects of these compounds on telomerase activity were evaluated in MCF7 and HDF cell lines using Telomeric Repeat Amplification Protocol and ELIZA assay. The highest increase in telomerase activity was observed at 0.004 mg/ml of sapogenin by 26% ±20.5 in MCF7 cells, while in HDF cells in the same concentration telomerase activity decreased down to 47%±0.48 and the highest inhibition of telomerase activity was observed at 0.070 mg/ml of sapogenins from *Spirulina* by 68%±0.43. In conclusion, a compound could play a role as a telomerase activator in one cell line while it could play another role as a telomerase inhibitor in another cell line so introducing compounds as a telomerase inhibitor (anticancer) or as a telomerase activator (anti-aging) should be done with discreet.

## INTRODUCTION

*Spirulina platensis* is one of the most important edible microalgae and because of its rich micro- and macronutrient contents, has a potential of food supplement production and bioactive compounds extraction properties like anti-cancer, anti-aging, anti-bacterial, anti-viral, immune protective, anti-oxidant, etc [1, 2]. Telomeres protect the chromosomes from degeneration rearrangement and end to end fusion. During DNA replication the telomeric fragments were added to 3 ends of chromosomes by a reverse transcriptase enzyme that nominated telomerase. Telomeres and telomerase play an important role in the age-related diseases and cancer [3, 4]. Telomerase activity is high in cancer cell line whereas its activity is low in age-related diseases. Since telomerase discovery, it is a molecular target to cancer therapy while on the other hand delaying aging prosses [5].

## MATERIALS AND METHODS

*Spirulina platensis* was obtained from (ABRII), Agricultural Biotechnology Research Institute of Iran and cultured in pH=9.5-10 at 30±3°C with an exposure of 4 kilolux light intensity. The culture was carried out within a shaker. Biomass was collected, dried and stored at 2-4°C [6]. Saponins were extracted as explain by Majinda, briefly, 0.05 g of methanolic extract dissolve in a mixture of 5 ml of distill water and 5 ml of butanol. Saponins separated in the organic phase [7]. To obtain sapogenins, 2 mg of saponins were hydrolyzed with 19 ml of 2N HCl in 50% aqueous methanol at 100°C for 2 h. The hydrolyzed mixture was extracted with 15 ml of ethyl acetate. Organic phase that containing sapogenins was collected and dried at 40°C [8]. 

MCF7 and HDF cell lines obtained from Pasteur Institute of Iran. The cells were cultured in RPMI-1640 medium, containing 10% Fetal Bovine Serum and 1%penicillin-streptomycin, (Gibco) under anaerobic incubation of 5% CO_2_ within CO_2_ incubator at 37°C [9]. The MCF7 and HDF cell lines were treated with final concentration of 0.004, 0.070 and 0.500 mg/ml of sapogenins from *spirulina* for 24h. All experiments were repeated three times. All experiments were repeated three times. Telomerase activity was evaluated with Telomerase Repeated Amplification Protocol and Enzyme-Linked Immunosorbent assay for each reaction by using Telo TAGGG telomerase PCR ELISA kit, (Roche(. Untreated HDF and MCF7 cells were used as controls. Increase or decrease of telomerase activity were compared with controls and reported.

## RESULTS AND DISCUSSION

**Figure 1:**
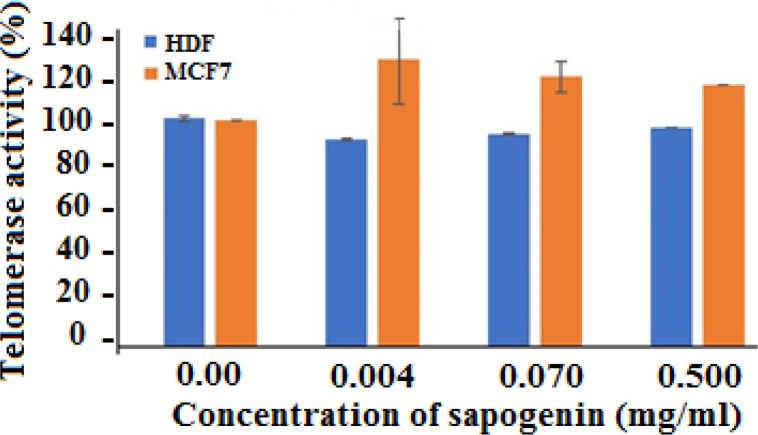
Telomerase activity after treatment HDF and MCF7 cell lines with different concentration of sapogenin from *S. platensis*

Our finding showed sapogenins from *S. platensis* have different effects on telomerase activity in different cell lines. As in Yurtcu *et al*. study has been reported Silymarin inhibited telomerase activity in HepG2 cell line [10]. while according to study of Parzonko *et al.* Silymarin increase telomerase activity in endothelial progenitor cells [11]. In our study telomerase activity was inhibited after treatment with sapogenins in HDF cells. The highest inhibition of telomerase activity in HDF cells was observed at 0.070 mg/ml of sapogenins by 68%±0.43 while in MCF7 at the same concentration increment of telomerase activity observed by 19.1%±6.7. In MCF7 cells, treatment with other concentrations of sapogenins increased telomerase activity as well (Fig. 1).

TA65 (cycloastragenol) is a sapogenin extracted from the root of *Astragalus membranaceus* that is known as an anti-aging agent. TA65 can increase average telomer length by enhancement in telomerase activity in keratinocytes, ﬁbroblasts, and immune cells in culture [12]. whereas in our study sapogenins from *S. platensis* inhibit telomerase activity in HDF. 

 Telomerase has a major protein subunit that nominated as TERT [13]. One of the reasons for different effects of the same compounds on telomerase activity can be alternative telomerase TERT mRNA splicing. This phenomenon in the expression of TERT can produce different variants of TERT gene. In TERT subunit ten different splice variants have been identiﬁed. TERT transcriptional control play a vital role in the complex regulation of telomerase activity [14].

Another reason can be related to the presence of different protein kinases in different cells. 

protein kinase C (PKC) at post-transcription can regulate the enzyme activity by kinase phosphorylation. Revocable phosphorylation of TERT catalytic subunit in speciﬁc serine/ threonine or tyrosine residues that occur in posttranslational regulation of telomerase activity, can influence on posttranslational regulation of telomerase activity. Numerous reports prove the role of PKC isoforms in telomerase regulation. It has been realized that telomerase in human breast cancer cells is controlled by PKC α through phosphorylation of hTERT while in nasopharyngeal cancer cells and peripheral T lymphocytes, during T-cell activation PKC ζ has been described to regulate telomerase activity. The research on head and neck cancer cells showed that expression of PKC isoenzymes α, β, δ, Ɛ and ζ and TERT phosphorylation was related with increment of telomerase activity in head and neck tumor cells. These results illustrated that telomerase is regulated by various PKC isoenzymes, that maybe depending on the cell type or cell position [14, 15]. A compound may directly, effect on protein kinase C so that indirectly affects telomerase activity.

To conclude, evaluation of telomerase activity is important in age-related diseases and cancer. One promising approach to treat cancer is the use of telomerase inhibitors. Using telomerase activators for improved condition of elderly patients and for the treatment of age-related diseases is also recommended. However, it should be noted that the effects of a compound on telomerase in a cell line could be inhibitory, whereas in another cell it might increase telomerase activity. Therefore, introducing compounds as a telomerase inhibitor (anticancer) or as a telomerase activator (anti-aging) should be done with cautious. 
